# Inflammatory Disease Affecting the Central Nervous System in Dogs: A Retrospective Study in England (2010–2019)

**DOI:** 10.3389/fvets.2021.819945

**Published:** 2022-01-27

**Authors:** Rita Gonçalves, Steven De Decker, Gemma Walmsley, Sarah Butterfield, Thomas W. Maddox

**Affiliations:** ^1^Department of Small Animal Clinical Science, Small Animal Teaching Hospital, University of Liverpool, Liverpool, United Kingdom; ^2^Department of Musculoskeletal and Ageing Science, Institute of Lifecourse and Medical Sciences, University of Liverpool, Liverpool, United Kingdom; ^3^Department of Clinical Science and Services, Royal Veterinary College, University of London, London, United Kingdom

**Keywords:** canine, central nervous system (CNS), meningoencephalitis of unknown origin (MUO), SRMA, infection

## Abstract

The epidemiology of inflammatory diseases affecting the central nervous system (CNS) in dogs is largely unknown. We aimed to report the relative proportion of different causes of inflammatory disease affecting the CNS in dogs and identify predictors for infectious vs. immune-mediated conditions and predictors for the most common diseases affecting the brain and the spinal cord. This was a retrospective cohort study over a 10-year period in 2 referral institutions using multivariable and multinomial logistic regression for identification of risk factors. In total, 1,140 client-owned dogs diagnosed with inflammatory disease affecting the CNS were included. Fifteen different diagnoses were identified, with immune-mediated (83.6%) disease being more common than infectious conditions (16.4%). The most common immune-mediated conditions diagnosed were meningoencephalitis of unknown origin (47.5%) and steroid-responsive meningitis–arteritis (30.7%), and the most common infectious conditions were discospondylitis (9.3%) and otogenic intracranial infection (2.2%). Older age (*p* < 0.001, OR = 1.019, 95% CI: 1.014–1.024), higher body weight (*p* < 0.001, OR = 1.049, 95% CI: 1.025–1.074), male sex (*p* = 0.009, OR = 1.685, 95% CI: 1.141–2.488), longer duration of the clinical signs before presentation (*p* < 0.001, OR = 1.011, 95% CI: 1.006–1.017), progressive nature of the clinical signs (*p* < 0.001, OR = 2.295, 95% CI: 1.463–3.599), identification of a possibly associated preceding event (*p* = 0.0012, OR = 1.93, 95% CI: 1.159–3.213), and hyperesthesia on presentation (*p* < 0.001, OR = 2.303, 95% CI: 1.528–3.473) were associated with a diagnosis of infectious diseases. Our data shows that immune-mediated diseases are more common than infectious conditions as a cause for inflammatory CNS disease in dogs. The risk factors for the most common diagnoses were identified from signalment, history, and findings of the physical and neurological examinations to give valuable information that can guide clinicians with their investigations.

## Introduction

Inflammatory disease affecting the central nervous system (CNS) in dogs can be due to infectious or immune-mediated causes (1–3). Infectious conditions include diseases caused by bacterial, viral, protozoal, rickettsial, fungal, parasitic, and algal agents (1–6). On the other hand, several idiopathic non-infectious meningoencephalomyelitides (NIMEs) have been described, including steroid-responsive meningitis–arteritis (SRMA) (7, 8), eosinophilic meningoencephalitis (9), granulomatous meningoencephalomyelitis (GME) (10–12), necrotizing meningoencephalomyelitis (NME) (10–14), necrotizing leucoencephalitis (NLE) (15, 16), idiopathic generalized tremor syndrome (IGTS) (17, 18), and idiopathic hypertrophic pachymeningitis (19). Due to the difficulty of distinguishing between GME, NME, and NLE without histopathological confirmation (which is rarely sought *in vivo*), the term meningoencephalomyelitis of unknown origin (MUO) is often used to describe cases for which any of these three conditions is suspected (20).

A definitive diagnosis, in most cases, would require a histopathological examination of the brain and/or spinal cord tissue, and so in clinical practice, the diagnosis of inflammatory CNS diseases relies on a cautious review of the clinical data. Clinical diagnosis is based on a combination of signalment, neurological deficits detected on examination, magnetic resonance imaging (MRI) findings, and results of cerebrospinal fluid (CSF) analysis and of infectious disease titers and antigen tests (20, 21). Treatment of infectious conditions relies on the use of specific medications against the infectious agent identified and, when dealing with bacterial diseases, preferably guided by culture and susceptibility results (2, 3). Conversely, treatment of immune-mediated conditions consists of immunosuppression, generally using corticosteroids, and often other immunosuppressive drugs (22).

It is currently suspected that NIMEs are more common than infectious causes, although the actual prevalence of any of these conditions is still undetermined in dogs (21). Our aim was therefore to report the proportion of the different types of inflammatory diseases affecting the CNS in England and to identify predictors for infectious and immune-mediated causes. As the causes for intracranial and spinal cord CNS inflammatory diseases are very different, we also aimed to identify predictors for the most common conditions affecting the brain and the spinal cord individually.

## Materials and Methods

Dogs diagnosed with any form of inflammatory CNS disease at the Small Animal Teaching Hospital (SATH) of the University of Liverpool and the Queen Mother Hospital for Animals (QMHA) of the Royal Veterinary College between January 2010 and December 2019 were identified through their respective hospital databases. Ethical approval for use of data was granted by the Ethics Committee of the University of Liverpool (VREC906).

Inclusion required that all clinical information required for statistical analysis was available for review and that historical findings, clinical signs, and clinicopathological and imaging findings allowed for a highly probable diagnosis; histopathological confirmation was used where available. A diagnosis of MUO required that dogs had to be older than 6 months of age, showed multiple, single, or diffuse intra-axial hyperintensities on T2-weighted MR images, had mononuclear pleocytosis on CSF analysis [more than 5 total nucleated cell count (TNCC)/μl, with more than 50% mononuclear cells], and with negative *Toxoplasma gondii* and *Neospora caninum* titers (20). Dogs were excluded if no pleocytosis was found on CSF analysis, with the exception of dogs with signs of raised intracranial pressure (ICP) on imaging studies, in which case CSF collection was not performed.

The following data were extracted from the medical records of the animals: age, sex, neuter status, breed, year of presentation, season of the year, onset, duration and progression of the clinical signs, description of any previous episode with similar clinical signs, identification of a possibly associated preceding event, presence of hyperthermia (>39.5°C) on presentation, presence of hyperesthesia on presentation, neurological deficits identified on presentation, and survival to discharge. Onset of the clinical signs was categorized as acute (<24 h), subacute (between 24 and 72 h), and chronic (more than 72 h).

MRI was performed in 958 cases using a 1.5-T scanner (Philips Ingenia CX, Philips Healthcare) and in 182 cases using a 1-T scanner (Siemens Magnetom, Siemens Healthcare), and the following information was recorded: if there were signs of raised intracranial pressure (identification of foramen magnum herniation, transtentorial herniation, and/or effacement of cerebral sulci) and whether there was one or multiple lesions seen. When cerebrospinal fluid analysis was performed, the following information was recorded: TNCC, protein concentration, and differential cell count. The results of the CSF analysis were considered abnormal when the protein concentration was >0.30 g/L (cerebellomedullary cistern collection) or >0.45 g/L (lumbar collection) or when the TNCC was >5 cells/μl. Additional test results, including synovial fluid analysis, infectious disease titers, and CSF culture results, were recorded when available.

Information regarding survival to hospital discharge was recorded for all cases, and data on long-term survival was collected from the records when available; the referring veterinary surgeons and pet owners were not contacted for the purposes of this study.

### Statistical Analysis

Statistical analysis was performed using the software SPSS 22.0 (SPSS Inc., Chicago, Illinois, USA). Continuous data were assessed for normality using the Shapiro–Wilk test. Descriptive statistics are reported for continuous variables using mean (standard deviation) for approximately normally distributed variables and median (interquartile range) for variables with skewed distributions, and frequencies [with 95% confidence intervals (CI) where appropriate] are reported for categorical variables.

Univariable binary logistic regression was performed to identify clinical variables associated with either an underlying immune-mediated or infectious cause among all cases. Any independent variable demonstrating some association on preliminary univariable analysis (*P* < 0.2) was considered for inclusion in a multivariable model. Multivariable models were constructed with a manual backwards stepwise removal approach; variables with *P* < 0.05 were retained as statistically significant. The association between the underlying immune-mediated or infectious cause and survival to discharge was evaluated using the chi-square test.

Multinomial logistic regression was then used to further identify clinical variables associated with the three largest groups of diseases affecting the brain and the spinal cord separately. Inflammatory conditions affecting the brain were grouped in 3 categories: MUO (including the focal, disseminated, and optic forms), immune-mediated causes other than MUO (including idiopathic generalized tremor syndrome, eosinophilic meningoencephalitis of unknown origin, and idiopathic hypertrophic meningitis), and all infectious diseases. Inflammatory conditions affecting the spinal cord were grouped in 3 categories: SRMA, meningomyelitis of unknown origin, and all infectious causes. Similarly, univariable multinomial logistic regression was performed initially to identify clinical variables associated with the different brain and spinal cord disease categories, using MUO as the reference category for intracranial disease and SRMA as the reference category for spinal disease. Any independent variable demonstrating some association on preliminary univariable analysis (*P* < 0.2) was considered for inclusion in a multivariable model. Multivariable models were constructed with a manual backwards stepwise removal approach; variables with *P* < 0.05 were retained as statistically significant.

Variables included in the univariable analysis included age (months), body weight (kg), sex, neuter status, breed category, year of presentation, season of the year, center, onset, duration (in days) and progression of the clinical signs (defined as the development of new signs), description of a previous episode with similar clinical signs, identification of a possibly associated preceding event, presence of hyperthermia on presentation, presence of hyperesthesia on presentation, neurolocalization [categorized as forebrain, brainstem, cerebellum, central vestibular (used when there were insufficient signs to localize as brainstem or cerebellum), optic nerves/chiasm, C1–C5, C6–T2, T3–L3, and L4–S3 spinal cord segments, multifocal or no neurological deficits when only pain was identified], MRI abnormalities (one or multiple lesions identified), and CSF analysis findings (TNCC and protein concentration). Due to the significant number of cases in which MRI and/or CSF analysis had not been performed, these variables were not included in the multivariable analysis. For statistical analysis, the breeds were categorized according to the American Kennel Club guidelines (organized by the original type of work each breed was developed to do) into the following groups: sporting, hound, working, terrier, toy, herding, and non-sporting.

## Results

A total of 1,140 client-owned dogs diagnosed with inflammatory disease affecting the CNS were included in the analysis, with 450 dogs presented to the SATH and 690 presented to the QMHA. A total of 36 dogs that had been initially identified with a suspected diagnosis of MUO were excluded due to having normal TNCC on CSF analysis; 18 of these dogs were from breeds with reported necrotizing encephalitis and long duration of clinical signs.

A total of 15 different diagnoses were identified (Table 1), with immune-mediated diseases (83.6%) being considerably more common than infectious conditions (16.4%). In 604 dogs, the clinical signs suggested an intracranial neurolocalization (Table 2), while 536 dogs presented signs of spinal cord diseases (Table 3). An immune-mediated disease was diagnosed in 88.2% of dogs with intracranial neurolocalization, and 78.8% of dogs were diagnosed with spinal neurolocalization.

**Table 1:** Proportion of the causes of inflammatory disease affecting the central nervous system in 1,140 dogs.

**Suspected diagnosis**	**Frequency**	**Percentage (%)**	**95% CI**
Meningoencephalomyelitis of unknown origin (MUO)^a^	541	47.5	44.7–50.4
Steroid responsive meningitis-arteritis (SRMA)^b^	350	30.7	28–33.2
Discospondylitis	106	9.3	7.7–11.1
Idiopathic generalized tremor syndrome	39	3.4	2.5–4.5
Otogenic intracranial infection	25	2.2	1.4–3.1
Empyema^c^	16	1.4	0.7–2.1
Eosinophilic meningoencephalitis	13	1.1	0.6–1.8
Bacterial meningoencephalitis	12	1.1	0.5–1.7
Neosporosis	12	1.1	0.5–1.7
Idiopathic hypertrophic meningitis	11	1	0.4–1.6
Angiostrongylosis	5	0.4	0.1–0.8
Fungal encephalitis	3	0.3	0–0.6
Distemper	3	0.3	0–0.6
Toxoplasmosis	2	0.2	0–0.4
Rickettsial meningoencephalitis	2	0.2	0–0.4

*CI, confidence interval*.

a *Within the MUO category, 73 dogs had meningomyelitis and 16 dogs had optic neuritis*.

b *SRMA was associated with concurrent immune-mediated polyarthritis in 31 cases*.

c *Empyema affected the brain in 12 cases and the spine in 4 cases*.

**Table 2:** Descriptive statistics for the 604 dogs with inflammatory disease affecting the brain.

	**MUO** **(*n* = 468)**	**Other immune-mediated causes** **(*n* = 63)**	**Infectious causes** **(*n* = 73)**
Median age (IQR)	48 months (51)	27 months (39)	58 months (61)
Median body weight (IQR)	8 kg (7)	9.9 kg (11.3)	15.6 kg (17.8)
**Sex**			
Female	259 (55.4%)	36 (57.1%)	24 (32.9%)
Male	209 (44.6%)	27 (42.9%)	49 (67.1%)
**Neuter status**			
Intact	183 (39.1%)	22 (34.9%)	31 (42.5%)
Neutered	285 (60.9%)	41 (65.1%)	42 (57.5%)
**Breed categories**			
Crossbreeds	66 (14.2%)	15 (23.8%)	6 (8.2%)
Sporting	48 (10.3%)	8 (12.7%)	19 (26%)
Hound	8 (1.7%)	17 (27%)	8 (11%)
Working	14 (3%)	1 (1.6%)	12 (16.5%)
Terrier	95 (20.3%)	13 (20.6%)	5 (6.8%)
Toy	185 (39.6%)	7 (11.1%)	12 (16.5%)
Herding	7 (1.5%)	1 (1.6%)	2 (2.7%)
Non-sporting	44 (9.4%)	1(1.6%)	9 (12.3%)
Previous similar episode	31 (6.6%)	1 (1.6%)	9 (12.3%)
Possibly associated preceding event	31 (6.6%)	13 (20.6%)	7 (9.6%)
**Onset of clinical signs**			
Acute	156 (33.4%)	15 (23.8%)	26 (35.6%)
Subacute	110 (23.6%)	17 (27%)	14 (19.2%)
Chronic	201 (43%)	31 (49.2%)	33 (45.2%)
Median duration of clinical signs (IQR)	7 days (12)	7 days (10)	6 days (19)
Progression of clinical signs	388 (82.9%)	54 (85.7%)	65 (89%)
Hyperthermia on presentation	22 (4.7%)	16 (25.4%)	16 (22%)
Hyperesthesia on presentation	152 (32.5%)	10 (15.9%)	27 (37%)
Median CSF TNCC (IQR)	36 cells/μl (208)	15 cells/μl (63)	201 cells/μl (2,409)
Median CSF protein concentration (IQR)	0.47 g/L (0.73)	0.21 g/L (0.24)	0.69 g/L (1.54)
Survival to discharge	381 (81.4%)	62 (98.4%)	62 (84.9%)

*CSF, cerebrospinal fluid; IQR, interquartile range; MUO, meningoencephalomyelitis of unknown origin; TNCC, total nucleated cell count*.

**Table 3:** Descriptive statistics for the 536 dogs with inflammatory disease affecting the spinal cord.

	**SRMA**	**MUO**	**Infectious causes**
	**(*n* = 350)**	**(*n* = 73)**	**(*n* = 113)**
Median age (IQR)	11 months (8)	52 months (59)	76 months (91)
Median body weight (IQR)	14.1 kg (11.1)	9.8 kg (14.6)	22 kg (15.3)
**Sex**			
Female	146 (41.8%)	41 (56.2%)	42 (35.7%)
Male	203 (58.2%)	32 (43.8%)	71 (64.3%)
**Neuter status**			
Intact	203 (57.5%)	26 (35.6%)	33 (37.2%)
Neutered	150 (42.5%)	47 (64.4%)	51 (62.8%)
**Breed categories**			
Crossbreeds	68 (19.6%)	13 (18.1%)	16 (14.2%)
Sporting	77 (22.2%)	5 (7%)	32 (28.3%)
Hound	86 (24.8%)	4 (5.6%)	6 (5.3%)
Working	38 (10.9%)	7 (9.7%)	24 (21.2%)
Terrier	23 (6.6%)	7 (9.6%)	11 (9.7%)
Toy	28 (8.1%)	27 (37.5%)	3 (2.7%)
Herding	24 (6.9%)	1 (1.4%)	2 (1.8%)
Non-sporting	3 (0.9%)	8 (11.1%)	19 (16.8%)
Previous similar episode	81 (23.2%)	4 (5.4%)	14 (12.4%)
Possibly associated preceding event	68 (19.4%)	9 (12.3%)	25 (22.1%)
**Onset of clinical signs**			
Acute	89 (25.4%)	17 (23.3%)	17 (15%)
Subacute	135 (38.6%)	16 (21.9%)	22 (19.5%)
Chronic	126 (36%)	40 (54.8%)	74 (65.5%)
Median duration of clinical signs (IQR)	4 days (5)	7 days (18)	13 days (27)
Progression of clinical signs	95 (27.1%)	68 (93.2%)	79 (69.9%)
Hyperthermia on presentation	289 (82.6%)	9 (12.3%)	28 (24.8%)
Hyperesthesia on presentation	341 (97.4%)	46 (60.3%)	104 (92%)
Median CSF TNCC (IQR)	249 cells/μl (1,035)	174 cells/μl (835)	3 cells/μl (7)
Median CSF protein concentration (IQR)	0.65 g/L (1.16)	1.27 g/L (2.89)	0.55 g/L (0.8)
Survival to discharge	348 (99.4%)	67 (91.7%)	105 (92.9%)

*CSF, cerebrospinal fluid; IQR, interquartile range; MUO, meningoencephalomyelitis of unknown origin; SRMA, steroid-responsive meningitis–arteritis; TNCC, total nucleated cell count*.

The cases presented evenly across the 10 years of study (between 7.7 and 12.7% each year) and throughout the seasons, with 23.3% presenting in winter, 25.3% in spring, 25.2% in summer, and 25.8% in autumn. Preceding events that may be associated with a condition were identified in 152 (13.3%) cases. The most commonly reported preceding events included a recent surgical procedure (63/152, 41.5%; the surgical procedure was neutering in 38 cases and spinal surgery in 5 cases), an infection focus (such as urinary tract infection or skin abscess) identified prior to the development of clinical signs (33/152, 21.7%), development of gastrointestinal signs prior to the onset of neurological deficits (27/152, 17.8%), vaccination within 2 weeks from the onset of clinical signs (20/152, 13.2%), and recent estrus or phantom pregnancy (10/152, 6.6%).

Magnetic resonance imaging was performed in 796 cases (in 576 dogs, MRI of the brain was performed; in 202 dogs, it was MRI of the spinal cord; and in 18 dogs, it was MRI of both the brain and spinal cord) and was abnormal in 692 cases. The cases that did not undergo MRI mostly comprised dogs diagnosed with SRMA and discospondylitis (which, in some cases, had undergone radiography and/or computed tomography); radiography was performed in 78 dogs and computed tomography in 63 dogs. In dogs with intracranial disease, 189/590 (32%) showed signs on MRI suggestive of raised intracranial pressure, and 371/590 (62.9%) had multiple lesions identified, while in dogs with a spinal disease, only 60/206 (29.1%) had multiple lesions identified. Cerebrospinal fluid analysis was performed in 903 cases, with identification of signs of suspected raised intracranial pressure on MRI precluding CSF collection in 102 dogs (Tables 2, 3). CSF was collected from the cerebellomedullary cistern in 713 cases and from the lumbar cistern in 106; in 84 dogs, CSF was collected from both sites. Serum titers of *Toxoplasma gondii* (IgG and IgM) and *Neospora caninum* (IgG) were performed in 408 cases and revealed positive titers for *T. gondii* in 23 cases and *N. caninum* in 12 cases; in 21 cases, they were suggestive of exposure, and the repeat titers were supportive of this. Polymerase chain reaction to identify different infectious agents (most commonly canine distemper virus—CDV, *T. gondii* and *N. caninum*) was performed on CSF from 195 dogs and confirmed CDV in 3 dogs, *N. caninum* in 5 dogs, and *Ehrlichia canis* in 2 dogs. Cerebrospinal fluid culture was performed in 70 cases, and culture of other tissues (blood, urine, and/or wounds) was performed in 165 cases. The bacterial cultures were positive in 78 dogs: 22 on urine culture, 19 on blood culture, 6 on intervertebral disc culture, 4 on CSF culture, 4 on wound culture, 1 on post-mortem tissue culture, and 22 on surgical sample culture (12 from ventral bulla osteotomy, 6 from spinal surgery, and 4 from intracranial surgery). Arthrocentesis and synovial fluid analysis were performed in 52 cases and were supportive of immune-mediated polyarthritis in 31 cases (all these dogs were also diagnosed with SRMA). Histopathological confirmation of diagnosis was achieved in 44 cases through post-mortem examination and in 7 cases through surgical biopsies.

One thousand twenty-four dogs (89.8%) survived to discharge. Long-term follow-up information at 1 year following diagnosis was available for 744 dogs, and at that time, 554 dogs (65.3%) were still alive.

### Binary Logistic Regression Results

On univariable analysis, age, body weight, sex, breed category, study center, year of presentation, onset, duration and progression of the clinical signs, identification of a possibly associated preceding event, presence of pyrexia on presentation, and presence of hyperesthesia on presentation showed some evidence of association (*P* < 0.2) with a diagnosis of an underlying immune-mediated or infectious cause. On multivariable analysis, older age, longer duration of the clinical signs before presentation, being male, progressive nature of the clinical signs, and identification of a preceding event were associated with a diagnosis of infectious diseases (Table 4). In breed categories, sporting and non-sporting breeds (with the French and English bulldogs as the most common breeds represented in the latter group) were associated with infectious causes. There was no association between survival to discharge nor survival at 12 months after diagnosis with a diagnosis of an underlying immune-mediated or infectious cause (*p* = 0.729 and *p* = 0.69, respectively).

**Table 4:** Final multivariable logistic regression model for risk factors associated with infectious vs. immune-mediated causes among all cases.

	**OR**	**95% CI**	***P*-value**
Age (months)	1.019	1.014–1.024	<0.001^a^
Body weight (kg)	1.049	1.025–1.074	<0.001^a^
Sex	1.685	1.141–2.488	0.009^a^
Duration of clinical signs (days)	1.011	1.006–1.017	<0.001^a^
Progression of clinical signs	2.295	1.463–3.599	<0.001^a^
Possibly associated preceding event	1.93	1.159–3.213	0.0012^a^
Hyperesthesia on presentation	2.303	1.528–3.473	<0.001^a^
**Breed categories**			
Crossbreeds	Ref	–	–
Sporting	2.198	1.143–4.229	0.018^a^
Hound	0.913	0.405–2.06	0.826
Working	2.043	0.911–4.579	0.083
Terrier	0.69	0.311–1.529	0.36
Toy	0.7	0.312–1.568	0.386
Herding	0.788	0.231–3.043	0.788
Non-sporting	6.255	2.981–13.126	<0.001^a^

*CI, confidence interval; OR, odds ratio*.

a *Statistically significant*.

### Multinomial Logistic Regression Results for Intracranial Disease

On univariable analysis, age, body weight, sex, breed category, identification of a possibly associated preceding event, duration of signs, description of a previous episode with similar clinical signs, neurolocalization, presence of pyrexia on presentation, and presence of hyperesthesia on presentation showed some evidence of association (*P* < 0.2) with a diagnosis of MUO, immune-mediated causes other than MUO, or infectious diseases in dogs presenting with intracranial disease.

Identification of multiple lesions on MRI was negatively associated both with immune-mediated causes other than MUO (*p* < 0.001, OR = 0.118, 95% CI: 0.05–0.277) and with infectious causes compared to dogs with MUO (*p* < 0.001, OR = 0.161, 95% CI: 0.092–0.282).

On multivariable analysis (Table 5), a previous episode with similar clinical signs reported, male sex, and working, hound, and sporting breeds showed a significant association with a diagnosis of infectious causes compared to MUO. The hound breeds were positively associated, while the non-sporting breeds were negatively associated with a diagnosis of immune-mediated causes other than MUO compared to MUO. Hyperthermia was associated both with a diagnosis of immune-mediated causes other than MUO and with infectious causes compared to dogs with MUO. A cerebellar neurolocalization was associated with a diagnosis of immune-mediated causes other than MUO compared to MUO, and a central vestibular neurolocalization was associated with a diagnosis of infectious causes compared to MUO.

**Table 5:** Multinomial logistic regression results evaluating associations between different variables and a diagnosis of MUO, other immune-mediated causes, or infectious causes among cases with intracranial disease.

	**Final diagnosis**	**OR (95%CI)**	***P*-value**
Sex	MUO	Ref	–
	Other IM causes	0.805 (0.386–1.681)	0.564
	Infectious causes	2.379 (1.248–4.408)	0.006^a^
**Breed categories**			
Crossbreeds	Ref	–	–
Sporting	MUO	Ref	–
	Other IM causes	0.798 (0.234–2.723)	0.718
	Infectious causes	5.571 (1.836–16.907)	0.002^a^
Hound	MUO	Ref	–
	Other IM causes	19.075 (5.408–67.286)	<0.001^a^
	Infectious causes	19.381 (4.762–78.875)	<0.001^a^
Working	MUO	Ref	–
	Other IM causes	0.742 (0.074–7.429)	0.8
	Infectious causes	18.136 (4.928–66.748)	<0.001^a^
Terrier	MUO	Ref	–
	Other IM causes	0.583 (0.193–1.765)	0.34
	Infectious causes	0.521 (0.129–2.108)	0.361
Toy	MUO	Ref	–
	Other IM causes	0.283 (0.088–0.911)	0.034^a^
	Infectious causes	0.873 (0.276–2.763)	0.818
Herding	MUO	Ref	–
	Other IM causes	0.921 (0.069–12.346)	0.95
	Infectious causes	5.911 (0.854–40.927)	0.072
Non-sporting	MUO	Ref	–
	Other IM causes	0.102 (0.01–1.043)	0.054
	Infectious causes	2.011 (0.553–7.312)	0.289
Previous similar episode	MUO	Ref	–
	Other IM causes	0.322 (0.032–3.293)	0.339
	Infectious causes	3.084 (1.13–8.417)	0.028^a^
Duration of clinical signs before presentation (days)	MUO	Ref	–
	Other IM causes	1.013 (1.001–1.024)	0.031^a^
	Infectious causes	1.014 (1.005–1.024)	0.003^a^
Hyperthermia on presentation	MUO	Ref	
	Other IM causes	3.1 (1.105–8.696)	0.032^a^
	Infectious causes	5.157 (2.164–12.29)	<0.001^a^
**Neurolocalization**			
No neurological deficits	Ref	–	–
Forebrain	MUO	Ref	–
	Other IM causes	1.588 (0.263–9.579)	0.614
	Infectious causes	1.047 (0.31–3.539)	0.941
Brainstem	MUO	Ref	–
	Other IM causes	1.292 (0.133–12.545)	0.825
	Infectious causes	1.519 (0.347–6.646)	0.579
Cerebellum	MUO	Ref	–
	Other IM causes	67.907 (11.75–392.18)	<0.001^a^
	Infectious causes	3.221 (0.674–915.338)	0.143
Central vestibular	MUO	Ref	–
	Other IM causes	1.403 (0.161–12.225)	0.759
	Infectious causes	5.369 (1.564–18.433)	0.008^a^
Optic nerves/chiasm	MUO	Ref	–
	Other IM causes	3.611 (0.424–30.75)	0.24
	Infectious causes	0.404 (0.038–4.261)	0.451
Multifocal	MUO	Ref	–
	Other IM causes	1.585 (0.284–8.841)	0.599
	Infectious causes	0.893 (0.277–2.885)	0.85

*CI, confidence interval; IM, immune-mediated; MUO, meningoencephalomyelitis of unknown origin; OR, odds ratio; ref, reference category*.

a *Statistically significant*.

### Multinomial Logistic Regression Results for Spinal Cord Disease

On univariable analysis, age, sex, neuter status, breed category, description of a previous episode with similar clinical signs, onset, duration, and progression of the clinical signs neurolocalization, presence of hyperthermia on presentation, and presence of hyperesthesia on presentation (*P* < 0.2) was associated with a diagnosis of SRMA, meningomyelitis of unknown origin, or infectious causes in dogs presenting with a spinal disease.

On multivariable analysis (Table 6), older age was positively associated with a diagnosis of both MUO and infectious causes compared to SRMA. Increased body weight was associated with a diagnosis of infectious causes compared to SRMA. Sporting breeds were negatively associated with a diagnosis of MUO compared to SRMA. Hound breeds were negatively associated with a diagnosis of both MUO and infectious causes compared to SRMA. Non-sporting breeds were associated with a diagnosis of infectious causes compared to SRMA. Presence of hyperesthesia on presentation was negatively associated with a diagnosis of MUO compared to SRMA, while hyperthermia was negatively associated with a diagnosis of both MUO and infectious causes compared to SRMA. A chronic onset of the clinical signs was associated with a diagnosis of both MUO and infectious causes compared to SRMA. All spinal neurolocalizations were positively associated with a diagnosis of MUO compared to SRMA, and the T3–L3 and L4–S3 spinal segments were also associated with a diagnosis of infectious causes compared to SRMA.

**Table 6:** Multinomial logistic regression results evaluating associations between different variables and a diagnosis of SRMA, meningomyelitis of unknown origin (MUO) or infectious causes amongst cases with spinal disease using SRMA as the reference category.

	**Final diagnosis**	**OR (95% CI)**	***P*-value**
Age (months)	SRMA	Ref	–
	MUO	1.029 (1.01–1.049)	0.003^a^
	Infectious causes	1.053 (1.036–1.071)	<0.001^a^
Body weight (kg)	SRMA	Ref	
	MUO	0.994 (0.918–1.077)	0.88
	Infectious causes	1.063 (1.006–1.123)	0.031^a^
**Breed categories**			
Crossbreeds	Ref	–	–
Sporting	SRMA	Ref	–
	MUO	0.061 (0.008–0.475)	0.008^a^
	Infectious causes	0.969 (0.263–3.573)	0.963
Hound	SRMA	Ref	–
	MUO	0.032 (0.002–0.469)	0.012^a^
	Infectious causes	0.05 (0.005–0.504)	0.011^a^
Working	SRMA	Ref	–
	MUO	0.234 (0.024–2.316)	0.214
	Infectious causes	0.782 (0.142–4.302)	0.777
Terrier	SRMA	Ref	–
	MUO	1.087 (0.132–8.944)	0.938
	Infectious causes	1.839 (0.292–11.576)	0.516
Toy	SRMA	Ref	–
	MUO	0.442 (0.048–4.043)	0.47
	Infectious causes	0.122 (0.011–1.411)	0.092
Herding	SRMA	Ref	–
	MUO	0.085 (0–44.842)	0.441
	Infectious causes	0.048 (0–15.36)	0.302
Non-sporting	SRMA	Ref	–
	MUO	0.552 (0.047–6.533)	0.638
	Infectious causes	14.159 (2.027–98.914)	0.008^a^
Hyperthermia on presentation	SRMA	Ref	–
	MUO	0.09 (0.022–0.363)	0.001^a^
	Infectious causes	0.139 (0.053–0.367)	<0.001^a^
Hyperesthesia on presentation	SRMA	Ref	–
	MUO	0.047 (0.005–0.44)	0.007^a^
	Infectious causes	0.352 (0.044–2.828)	0.326
**Onset of the clinical signs**			
Subacute	Ref	–	–
Acute	SRMA	Ref	–
	MUO	2.039 (0.299–13.912)	0.467
	Infectious causes	0.695 (0.137–3.518)	0.66
Chronic	SRMA	Ref	–
	MUO	7.983 (1.642–38.813)	0.01^a^
	Infectious causes	4.169 (1.13–15.388)	0.032^a^
Progression of the clinical signs	SRMA	Ref	–
	MUO	11.348 (2.526–30.97)	0.002^a^
	Infectious causes	2.931 (1.061–8.098)	0.038^a^
**Neurolocalization**			
No neurological deficits	Ref	–	–
C1–C5	SRMA	Ref	–
	MUO	362.291 (36.51–3,595.059)	<0.001^a^
	Infectious causes	2.064 (0.151–28.24)	0.587
C6–T2	SRMA	Ref	–
	MUO	31.459 (2.55–388.053)	0.001^a^
	Infectious causes	4.426 (0.419–46.736)	0.216
T3–L3	SRMA	Ref	–
	MUO	486.444 (41.354–5,721.931)	<0.001^a^
	Infectious causes	59.701 (5.871–607.11)	0.001^a^
L4–S3	SRMA	Ref	–
	MUO	574.41 (13.08–25,225.188)	0.001^a^
	Infectious causes	127.273 (3.723–4,350.853)	0.007^a^
Multifocal	SRMA	Ref	–
	MUO	404.147 (1.626–99,290.213)	0.033^a^
	Infectious causes	7.229 (0.032–1,624.233)	0.474

*CI, confidence interval; MUO, meningoencephalomyelitis of unknown origin; OR, odds ratio; ref, reference category; SRMA, steroid-responsive meningitis–arteritis*.

a *Statistically significant*.

## Discussion

Identifying the most common types of inflammatory disease affecting the CNS in dogs and their associated risk factors is important so that clinicians can develop appropriate differential diagnosis lists and be guided in diagnostic investigations. This study showed that immune-mediated conditions are considerably more common than infectious conditions, comprising over 80% of all inflammatory diseases affecting the CNS in a large population of dogs presenting to referral practices. The most common immune-mediated conditions diagnosed were MUO and SRMA (representing a combined total of 78.2% of all inflammatory diseases affecting the CNS in dogs), and the most common infectious conditions were discospondylitis and otogenic intracranial infection (accounting for a combined total of 11.5%). Older age, longer duration of the clinical signs before presentation, being male, progressive nature of the clinical signs, identification of a possibly associated preceding event, and presence of hyperesthesia on presentation were associated with a diagnosis of infectious diseases. Signalment, clinical history, and findings of the physical and neurological examinations were identified as different risk factors for the most common diagnoses of intracranial and spinal inflammatory conditions. Interestingly, there was no significant difference in short- or long-term survival between immune-mediated and infectious conditions.

In humans with encephalitis and meningitis, infectious causes have been reported as significantly more common than non-infectious ones, although between 20 and 60% of cases remain with an unknown diagnosis (23–25). Viral etiologies (most commonly enterovirus) are most common, followed by bacterial and sporadically fungal agents (23–25). Despite this, autoimmune encephalitis is being increasingly recognized, and it is thought that there are likely unidentified antibodies or other immune mechanisms explaining why many cases have an antibody-negative status (26). The etiology of MUO remains undetermined and likely has a multifactorial pathogenesis involving a combination of genetic factors and environmental triggers. Multiple attempts have been made to identify different infectious agents as possible triggers for MUO in dogs, but these have so far been unsuccessful (27–29). In contrast, myelitis in humans is significantly more likely to be non-infectious, with multiple sclerosis as the most common cause, while infectious causes account for 1–12% of cases (30, 31). In veterinary medicine, only one previous study has investigated an inflammatory disease affecting the CNS, and its findings were very different from those in this study, as infectious causes accounted for ~75% of the cases (1). In this study, canine distemper encephalitis (CDV) was the most common diagnosis (40%), followed by unclassified viral encephalitis (a group with non-suppurative meningoencephalomyelitis with no known virus detected on immunocytochemistry). This markedly contrasts with our findings as CDV was extremely rare in our population which was mostly vaccinated against CDV. It can also be speculated that some of the dogs diagnosed with unclassified viral encephalitis indeed had MUO, a less well-described entity at that time. In the previous study, most cases (over 80%) had post-mortem examination, likely affecting the proportion of dogs with less severe disease. Lastly, there is a 20-year time interval between both studies, so the difference in the results may also reflect temporal changes in the prevalence of the different diseases.

Our findings provide support for signalment being very useful in guiding the differential diagnosis list, with age, sex, body weight, and breed all showing significant associations with various diagnoses. Younger age was associated with SRMA, which was not unexpected as this condition has an age of onset typically between 6 and 18 months (3, 7, 32–35). Male sex was associated with a diagnosis of infectious conditions compared to MUO in dogs presenting with intracranial disease and also overall. A female predisposition in MUO has been reported in previous studies (10, 11, 14), but more recently, this finding has been disputed (21). In most immune-mediated diseases, no clear sex predisposition has been reported (18, 32–34), although a possibly slight male predisposition has been recorded in a cohort of dogs diagnosed with SRMA (35). In contrast, a male predisposition has been reported in discospondylitis (3, 36, 37) and possibly also in bacterial meningoencephalitis (4) and otogenic intracranial infection (38). In dogs presenting with intracranial disease, working, hound, and sporting breeds showed a statistically significant association with a diagnosis of infectious causes compared to MUO. It is well-known that small-breed dogs, particularly of toy and terrier breeds, are predisposed to MUO (10, 11, 21), so it was not surprising that these breeds were less likely to be diagnosed with infectious causes. Hound breeds were also positively associated with a diagnosis of immune-mediated causes other than MUO compared to MUO. This most likely reflects that sighthounds (a major representative of this breed group) might be predisposed to idiopathic hypertrophic meningitis (19), while similar breeds are affected by MUO and IGTS (18). In dogs presenting with a spinal cord disease, sporting and hound breeds were negatively associated with a diagnosis of MUO compared to SRMA, which was expected as these groups are predisposed to SRMA (7, 32–35). Interestingly, non-sporting breeds (the group mostly composed of English and French bulldogs) were positively associated with a diagnosis of infectious causes (most commonly discospondylitis) compared to SRMA; this predisposition has not been reported before.

Our results support that clinical history is very useful in guiding the clinician, as longer duration of the clinical signs before presentation, progressive nature of the clinical signs, and identification of a possibly associated preceding event were associated with infectious causes. The longer duration of the clinical signs before presentation in dogs with infectious causes is likely associated with the slower progression of some of the most common infectious conditions seen, namely, discospondylitis and otogenic intracranial infections, compared to dogs with MUO or SRMA (38, 39). The most commonly reported preceding events were surgical interventions and an infectious focus, and these have been associated with discospondylitis and bacterial meningoencephalitis (3, 4). In dogs presenting with a spinal disease, progression of the clinical signs was positively associated with a diagnosis of MUO and infectious causes compared to SRMA. In SRMA, two forms are reported, with a chronic protracted form possibly occurring following relapse of the acute disease and/or inadequate treatment (3, 7). Episodic and recurrent clinical signs are usually reported, and only rarely do neurological deficits develop in the chronic form, likely explaining the lack of progression of the clinical signs reported in the dogs with SRMA seen in this study.

The findings of the physical and neurological examinations also demonstrated usefulness in distinguishing between the different causes of inflammatory CNS diseases. Hyperthermia was positively associated with a diagnosis of immune-mediated causes other than MUO and with infectious causes compared to MUO in dogs presenting an intracranial disease. Hyperthermia was negatively associated with a diagnosis of both MUO and infectious causes compared to SRMA in dogs presenting with a spinal disease. This is not surprising as dogs with SRMA (7, 8), IGTS (18), and many infectious conditions affecting the CNS (3, 4, 38) are frequently reported with hyperthermia. As expected, different neurolocalizations were associated with different conditions. A cerebellar neurolocalization was significantly associated with a diagnosis of immune-mediated causes other than MUO compared to MUO, most likely related to the typical signs of cerebellar dysfunction seen in dogs with IGTS (particularly tremors and ataxia) (18). A central vestibular neurolocalization was significantly associated with a diagnosis of infectious causes compared to MUO in dogs presenting with an intracranial disease. It would seem most likely that this is related to many of the infectious causes of intracranial diseases being dogs diagnosed with otogenic intracranial infection, in which involvement of the cerebellomedullary region of the brainstem is most common (38). All spinal neurolocalizations were positively associated with a diagnosis of MUO compared to SRMA, and the T3–L3 and L4–S3 spinal segments were associated with a diagnosis of infectious causes compared to SRMA. This is an unsurprising finding as neurological deficits are not common with SRMA (7) but expected in MUO (21), and discospondylitis most commonly affects the lumbosacral and thoracolumbar regions (3, 36, 37, 37).

Only a small number of dogs with a suspected diagnosis of angiostrongylosis was identified. It is uncertain in these cases if the cause of the abnormalities detected on MRI was related to a direct effect of the migrating *Angiostrongylus vasorum* larvae or more likely due to the resulting coagulopathy seen with the infection, as both have been reported previously (40, 41). If it was the latter, these cases should not be considered true CNS infection, as the clinical signs would be associated with a vascular etiology rather than inflammatory. The authors felt that, without a histopathological evaluation, it would be impossible to rule out larvae migration, and even in the event of an indirect effect, when an infectious underlying cause would be present, dogs diagnosed with angiostrongylosis would be included. This may have introduced a small bias, but we felt that the information was relevant to the study.

The MRI findings were mostly non-specific, but the identification of multiple lesions on MRI was associated with MUO as expected (21). There were no seasonal, geographical (with one of the centers located in the north and the other in the south of England), or temporal variations over the decade studied identified in our data. It is unknown if our findings would be reproducible in other areas of the world with a higher prevalence of infectious agents, and so further studies evaluating regional differences are warranted.

There are several limitations to this study. The main limitation is the lack of a histopathological confirmation in the majority of cases, which makes misdiagnosis possible, and therefore the relative proportion of the diseases may be inaccurate. Most dogs (89.5%) survived to discharge, with many still alive at 12 months later. Thus, including a histopathological examination as part of the inclusion criteria would have reduced the sample size considerably and risked introducing much more significant bias to the results. Due to the lack of a histopathological confirmation, it was also not possible to distinguish between the subtypes of MUO, and therefore it was not possible to assess if more specific diagnoses would have shown different associations or impacted survival. Because of its retrospective nature, not all dogs underwent the same diagnostic investigations, and not all epidemiologic data were available for review. Dogs that did not undergo CSF analysis due to the identification of raised ICP on MRI were not excluded from the analysis, as this would have likely biased the data by excluding the most severely affected cases. Nonetheless, this may also have added bias if those dogs were misdiagnosed from the lack of that data. Cases with suspected MUO but with normal CSF analysis and/or normal MRI were excluded, and this may have falsely reduced the frequency of this disease, as normal findings in either of these modalities can be seen in MUO (10, 20, 42). It should also be considered that the presumptive diagnosis of many of these conditions is often based on a combination of factors, including signalment and clinical and imaging findings, and some of these were subsequently identified as associated with specific conditions; this was also likely a source of bias. Lack of data on MRI or CSF analysis in some cases raised difficulties in the application of multivariable statistical analysis—the reason is related to the fact that the different diseases diagnosed require different diagnostic tests, and so not all results are available for all animals. Imputation approaches would not be appropriate for the missing information, so the authors felt that it was more appropriate to exclude the MRI and CSF variables from the multivariable analyses.

Our data confirms that immune-mediated disease is more common than infectious conditions as a cause for an inflammatory CNS disease in dogs as previously suspected. Signalment, clinical history, and findings of the physical and neurological examinations were identified as different risk factors for the most common diagnoses, presenting valuable information which can help guide clinicians. Close to 90% of all dogs survived to discharge, with no significant difference in short- or long-term survival between immune-mediated and infectious conditions.

## Data Availability Statement

The raw data supporting the conclusions of this article will be made available by the authors, without undue reservation.

## Ethics Statement

The animal study was reviewed and approved by Ethics Committee of the University of Liverpool. Written informed consent was obtained from the owners for the participation of their animals in this study.

## Author Contributions

RG and GW were responsible for the conception of the study. Data acquisition was done by RG, SD, and SB. Statistical analysis, data analysis, and manuscript writing were performed by RG. TM provided statistical advice. GW, SD, and TM supervised the data collection and manuscript editing. All authors contributed to the article and approved the submitted version.

## Conflict of Interest

The authors declare that the research was conducted in the absence of any commercial or financial relationships that could be construed as a potential conflict of interest.

## Publisher's Note

All claims expressed in this article are solely those of the authors and do not necessarily represent those of their affiliated organizations, or those of the publisher, the editors and the reviewers. Any product that may be evaluated in this article, or claim that may be made by its manufacturer, is not guaranteed or endorsed by the publisher.
